# Skin autofluorescence is associated with inappropriate left ventricular mass and diastolic dysfunction in subjects at risk for cardiovascular disease

**DOI:** 10.1186/s12933-017-0495-9

**Published:** 2017-01-25

**Authors:** Chun-Cheng Wang, Yao-Chang Wang, Guei-Jane Wang, Ming-Yi Shen, Yen-Lin Chang, Show-Yih Liou, Hung-Chih Chen, An-Sheng Lee, Kuan-Cheng Chang, Wei-Yu Chen, Chiz-Tzung Chang

**Affiliations:** 1 Graduate Institute of Clinical Medical Science, China Medical University, No. 91, Hsueh-Shih Road, Taichung, 40402 Taiwan; 2 Division of Cardiology, Department of Internal Medicine, Taichung Tzuchi Hospital, The Buddhist Tzuchi Medical Foundation, Taichung, Taiwan; 3 Division of Cardiovascular Medicine, Department of Internal Medicine, China Medical University Hospital, Taichung, Taiwan; 4 Division of Cardiothoracic Surgery, Chang Gung Memorial Hospital Keelung Branch, Keelung, Taiwan; 5 Department of Medical Research, China Medical University Hospital, Taichung, Taiwan; 6 Department of Health and Nutrition Biotechnology, Asia University, Taichung, Taiwan; 7 Department of Biomedical Engineering, Chun Yuan Christian University, Taoyuan, Taiwan; 8 Formosan Blood Purification Foundation, Taipei, Taiwan; 9 Division of Nephrology, Department of Internal Medicine, China Medical University Hospital, Taichung, Taiwan; 10 Department of Medicine, Mackay Medical College, New Taipei, Taiwan; 11 College of Medicine, China Medical University, No. 91, Hsueh-Shih Road, Taichung, 40402 Taiwan; 12 Cardiovascular Research Laboratory, China Medical University Hospital, Taichung, Taiwan; 13 Graduate Institute of Basic Medical Science, China Medical University, No. 91, Hsueh-Shih Road, Taichung, 40402 Taiwan

**Keywords:** Advanced glycation end product, Skin autofluorescence, Inappropriate left ventricular mass, Diastolic dysfunction

## Abstract

**Background:**

Enhanced advanced glycation end products deposition within myocardial tissue may cause diastolic dysfunction. However, whether this is related to left ventricular hypertrophy or inappropriate left ventricular mass remains unclear.

**Methods:**

We prospectively enrolled 139 subjects at risk for cardiovascular diseases. We used echocardiography for measurements of left ventricular mass and cardiac systolic and diastolic functional parameters. An advanced glycation end product reader was applied for measurements of skin autofluorescence values. Comparisons of left ventricular mass and echocardiographic parameters between the higher and lower skin autofluorescence groups were analyzed.

**Results:**

Compared with the lower skin autofluorescence group, left ventricular mass index and the ratio of observed left ventricular mass/predicted left ventricular mass (oLVM/pLVM) was significantly higher in the higher skin autofluorescence group (61.22 ± 17.76 vs. 47.72 ± 11.62, P < 0.01, 1.62 ± 0.38 vs. 1.21 ± 0.21, P < 0.01). After adjustment for potential confounding factors, skin autofluorescence was an independent factor for left ventricular mass index (β = 0.32, P < 0.01) and the ratio of oLVM/pLVM (β = 0.41, P < 0.01). Skin autofluorescence ≥2.35 arbitrary unit predicted left ventricular hypertrophy at a sensitivity of 58.8%, and a specificity of 73.0% (P < 0.01). Skin autofluorescence ≥2.25 arbitrary unit predicted inappropriate left ventricular mass at a sensitivity of 71.1%, and a specificity of 83.9% (P < 0.01). Skin autofluorescence was positively correlated with E/E′, an indicator for diastolic dysfunction (r = 0.21, P = 0.01).

**Conclusions:**

Skin autofluorescence is a useful tool for detecting left ventricular hypertrophy, inappropriate left ventricular mass and diastolic dysfunction.

**Electronic supplementary material:**

The online version of this article (doi:10.1186/s12933-017-0495-9) contains supplementary material, which is available to authorized users.

## Background

Increased left ventricular mass (LVM) may develop as a consequence of chronic pressure overload. When the growth of LVM exceeds its need to compensate for cardiac workload, it is defined as inappropriate LVM [1]. Clinically, inappropriate LVM has been proposed as an independent risk factor for adverse cardiovascular outcomes in hypertensive subjects [1–4]. Although the clinical significance of inappropriate LVM has been well validated, a clinically useful marker for predicting inappropriate LVM, especially in subjects at moderate to high cardiovascular (CV) risks, is yet to be fully investigated.

Advanced glycation end products (AGEs) are molecules formed by a nonenzymatic reaction between a reducing sugar and an amine group of protein, or lipid, called “the Maillard reaction” [5]. Enhanced AGEs accumulation throughout the skin, cardiac, renal, and vascular tissues can be found in aging, diabetic, or renal failure patients [5]. In cardiac tissue, the AGEs may covalently cross-link with extracellular matrix (ECM), such as collagen, laminin, and elastin, which may lead to the development of myocardial stiffness, impaired myocardial relaxation, and diastolic dysfunction [6–8]. In addition to their cross-link with ECM, AGEs could also stimulate the expression of ECM genes coding for type IV collagen, laminin [9]. Treatment with AGEs breaker (ALT-711) in Streptozocin-induced diabetic rats has also been demonstrated in association with reduced left ventricular mass, reduced protein expression of collagen tissue growth factor and collagen III [10]. For these reasons, AGEs accumulation may be associated with increased LVM. Skin autofluorescence (AF) is a simple, non-invasive method to measure the tissue accumulation of AGEs, and its accuracy has been validated [11]. In addition, skin AF has been validated as a useful tool for predicting long-term adverse cardiovascular outcomes in subjects of diabetes [12], and renal failure [13].

This study has thus been designed to test the hypothesis that skin AF measurement could be a useful tool to detect left ventricular hypertrophy (LVH), or even, inappropriate LVM in subjects at risk for CV diseases.

## Methods

### Study design and subjects

We prospectively recruited 139 subjects at risk for CV diseases at the age between 40 and 80 years old whose condition were stable, and had regular medical visits to our outpatient department for over 1 year. The definition of risk for CV diseases included (1) at least two conventional CV risk factors [family history of premature coronary artery disease, male gender with an age ≥45 years, or female gender with an age ≥50 years, hypertension, diabetes mellitus (DM), hyperlipidemia, and smoking]; (2) any history of ischemic heart disease (IHD), cerebrovascular disease or peripheral arterial occlusive disease (PAOD). The exclusion criteria included subjects with dementia, impaired conscious level, bed ridden status, any histories of psychiatric diseases, liver cirrhosis, end-stage renal disease requiring renal replacement therapy, advanced stage of cancer, or alcoholism. Subjects with prior myocardial infarction, cardiac arrhythmia, cardiomyopathy, or moderate-to-severe valvular heart diseases were also excluded. We obtained baseline demographic data by chart review and patients’ interview. Laboratory tests were all sampled within 3 months before the study enrollment. Body mass index (BMI) was defined as the body weight (g) divided by the square of body height (m^2^). DM was defined as a plasma level of glycohemoglobin ≥6.5% or the use of hypoglycemic medications for over 6 months. Hypertension was defined as a series of at least three systolic blood pressure (SBP) measurements ≥140 mmHg or diastolic blood pressure (DBP) measurements ≥90 mmHg at office or the use of anti-hypertensive medications for over 6 months. Hyperlipidemia was defined as a plasma level of total cholesterol >200 mg/dL, low-density lipoprotein cholesterol >130 mg/dL, triglyceride >150 mg/dL, or the use of lipid-lowering medications for over 6 months. Estimated glomerular filtration rate (eGFR) (mL/min/1.73 m^2^), a proxy for renal function, was calculated according to the modification of diet in renal disease (MDRD) study equation. IHD was defined as the presence of angina symptoms with evidence of ischemia diagnosed by non-invasive stress testing, or invasive coronary angiography. Cerebrovascular disease was defined as any event of ischemic stroke or transient ischemic attack diagnosed by computed tomography or magnetic resonance imaging. PAOD was defined as an ankle-brachial index (ABI) <0.9 further confirmed by non-invasive duplex ultrasound, computed tomography, or invasive peripheral angiography. The institution’s ethics committee for research in human subjects has approved the study. (reference numbers: CMUH103-REC2-021). Informed consent was obtained from each patient, and the study protocol conforms to the ethical guidelines of the 1975 Declaration of Helsinki as reflected in a priori approval by the institution’s human research committee.

### Echocardiography

All study subjects were instructed to rest for 30 min and blood pressure was measured thereafter by using a mercury sphygmomanometer before the study. Then echocardiography was performed in a dimly lit room with the study subjects in a partial left decubitus position. The methods of measuring echocardiographic parameters were in accordance with the recommendations from previous guidelines [14, 15]. In brief, left ventricular internal dimensions (LVID), septal and posterior wall thickness (SWT and PWT) were measured from the parasternal long-axis window at the level of mitral valve leaflet tips by using M-mode echocardiography. From this, the observed LVM (oLVM) was calculated as follows [16]:

$${\text{LVM\,(g)}} = 0.8 \times \{1.04[{\text{LVIDd}} +{\text{PWTd}} + {\text{SWTd}})^{3}]\}-({\text{LVIDd}}^{3} +0.6 {\text{g}}$$

Left ventricular mass index (LVMI) was computed as normalization of LVM for height to the 2.7th power. Left ventricular hypertrophy (LVH) was defined as LVMI ≥51 (g/m^2.7^) in men, and LVMI ≥47 (g/m^2.7^) in women [17]. Left ventricular volumes at end-diastolic (EDV) and end-systolic phase (ESV) were measured using the biplane method of disks (modified Simpson’s rule). From this, the stroke volume was generated and the left ventricular ejection fraction (LVEF) was calculated as follows:

$${\text{LVEF}} (\%) = ({\text{EDV}}-{\text{ESV}})/{\text{EDV}}\times 100\%$$

Theoretical individual LVM value predicted for sex, body size and cardiac workload was calculated as follows:

$${\text{Stroke work }}\left( {{\text{SW}}} \right){\text{ }}\left( {{\text{gram}} - {\text{meters}}/{\text{beat}}} \right) = {\text{ cuff systolic BP}} \times {\text{stroke volume}} \times 0.0{\text{144}};$$

$${\text{Predicted LVM }}\left( {{\text{pLVM}}} \right) = {\text{ 55}}.{\text{37 }} + {\text{ 6}}.{\text{64}}\times {\text{height}} \left( {{\text{m}}^{{{\text{2}}.{\text{7}}}} } \right) + {\text{ }}0.{\text{64}} \times {\text{SW }}\left( {{\text{gram}} - {\text{meters}}/{\text{beat}}} \right)- 18.07 \times {\text{gender}}$$

where male = 1, and female = 2. Appropriateness of LVM was defined as the ratio of oLVM indexed to the pLVM. The ratio of oLVM divided by pLVM was defined as inappropriate when there was an excess of >28% from the predicted value (oLVM/pLVM >128%) [4].

To further investigate the diastolic function, pulse-wave Doppler echocardiography was performed by placing a sample volume (1–3 mm) between the tips of the mitral valve in the apical four-chamber view during diastole [15]. Peak E (early diastolic) and A (late diastolic) mitral inflow velocities, the E/A ratio, deceleration time (DT) of early mitral filling velocity were derived. By applying pulsed-wave tissue Doppler echocardiography in the apical four-chamber view to acquire mitral annular velocity at the septal portion, E′ (early diastolic) annular velocity and A′ (late diastolic) annular velocity at the septal portion were derived. Thus, the E/E′, a proxy for mitral filling pressure, was generated.

### Skin AF as an estimation for tissue AGEs accumulation

The amount of skin AGE accumulation was estimated by measuring skin AF with an AGE reader (DiagnOptics Technologies BV, Groningen, the Netherlands). The AGE reader uses the fluorescence properties of some AGEs to estimate the accumulation of AGEs in skin. The AGE reader illuminated an ultraviolet light with wavelengths between 300 and 420 nm. The emitted light with wavelengths between 420 and 600 nm, and the reflected excitation light with wavelengths between 300 and 420 nm were measured with a spectrometer. Skin AF was measured as the ratio of the average light intensity between the emitted light and the reflected excitation light, multiplied by 100, and expressed as arbitrary units (AU). The protocol of skin AF measurement has been described elsewhere [11]. The intra-individual error percentage of repeat skin AF measurements taken within a day was 5.03%, and for seasonal variation was 5.87% [11].

### Statistical methods

Differences of continuous variables and categorical variables were compared with Student’s unpaired t test, and the Chi square test, respectively. The relations between skin AF, appropriateness of LVM, and echocardiographic parameters were analyzed with Pearson’s correlation test. Univariate linear regression analysis was applied to evaluate factors associated with LVMI and appropriateness of LVM. Factors with P value <0.05 were selected and included in the stepwise multivariate linear regression analysis to determine independent factors. Receiver operating characteristic (ROC) curve analysis was applied to determine the optimal skin AF value that best predicted LVH and inappropriate LVM.

## Results

### Participant characteristics

**Fig. 1:**
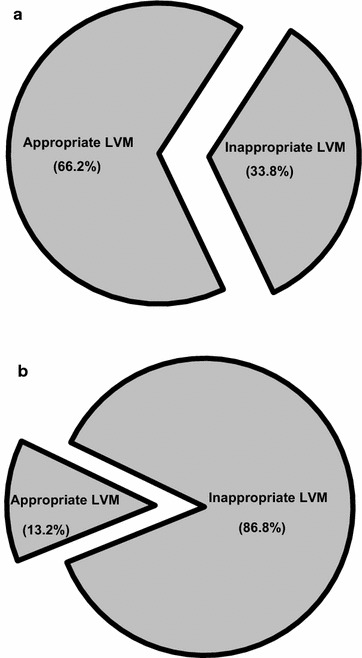
Proportions of inappropriate left ventricular mass in **a** lower skin autofluorescence group and in **b** higher skin autofluorescence group are depicted

All study subjects were divided into two groups according to the measured skin AF above or below the median value. Table 1 displays the baseline demographics and the comparisons between the higher skin AF group and the lower skin AF group. Compared with the lower skin AF group, the higher one was significantly older, and had higher proportions of IHD, and lower DBP. In the higher skin AF group, the proportion of inappropriate LVM is significantly higher than in the lower skin AF group (86.8 vs. 33.8%; χ^2^ P < 0.01). The results are in Fig. 1a, b.

**Table 1:** Baseline demographics of the study group

	Skin AF ≤2.2 (n = 71)	Skin AF >2.2 (n = 68)	P value
Age (years)	61.34 ± 11.34	66.57 ± 8.38	0.01
Gender (M/F)	41/30	43/25	0.51
Skin AF (A.U.)	1.90 ± 0.27	2.71 ± 0.31	0.01
BMI (kg/m^2^)	26.50 ± 3.86	26.05 ± 3.25	0.74
SBP (mmHg)	142.36 ± 14.83	139.06 ± 16.81	0.22
DBP (mmHg)	80.80 ± 13.57	75.41 ± 9.46	0.01
PP (mmHg)	61.92 ± 12.70	63.94 ± 14.64	0.39
Hypertension, n (%)	59 (83.10)	57 (83.82)	0.91
DM, n (%)			0.06
None, n (%)	50 (70.42)	40 (58.82)	
OHA, n (%)	20 (28.17)	21 (30.88)	
RI, n (%)	1 (1.41)	7 (10.29)	
Hyperlipidemia, n (%)	57 (80.28)	52 (76.47)	0.59
Stroke, n (%)	3 (4.23)	7 (10.29)	0.17
IHD, n (%)	9 (12.68)	21 (30.88)	0.01
PAOD, n (%)	1 (1.41)	1 (1.47)	0.98
eGFR (mL/min/1.73 m^2^)	66.64 ± 17.77	62.06 ± 22.73	0.19
Antiplatelets, n (%)	48 (67.61)	43 (63.24)	0.59
β-Blockers, n (%)	25 (35.21)	34 (50.00)	0.08
CCBs, n (%)	29 (40.85)	33 (48.53)	0.36
ACEIs/ARBs, n (%)	49 (69.01)	43 (63.24)	0.47
Statins, n (%)	40 (56.34)	41 (60.29)	0.64
Smoking			0.06
Never, n (%)	55 (77.46)	40 (58.82)	
Ever, n (%)	10 (14.08)	19 (27.94)	
Active, n (%)	6 (8.45)	9 (13.24)	

*Skin AF* skin autofluorescence, *AU* arbitrary unit, *BMI* body mass index, *SBP* systolic blood pressure, *DBP,* diastolic blood pressure, *PP* pulse pressure, *DM* diabetes mellitus, *OHA* oral hypoglycemic agent, *RI* regular insulin, *IHD* ischemic heart disease, *GFR* glomerular filtration rate, *CCBs* calcium channel blockers, *ACEIs* angiotensinogen converting enzyme inhbitors, *ARBs* angiotensin II type I receptor blockers

### Echocardiographic parameters

Comparisons of echocardiographic parameters between the higher skin AF group and the lower skin AF group are displayed in Table 2. Compared with the lower skin AF group, the higher one has significantly lower LVEF, indicating a poorer systolic performance. In addition, the higher skin AF group had significantly higher left atrial (LA) diameter, lower early diastolic peak velocity at septal portion of mitral annulus, and higher E/E′ ratio, indicating more diastolic dysfunction. Both the LVMI and the ratio of oLVM indexed to the pLVM were significantly higher in the higher skin AF group than in the lower skin AF group.

**Table 2:** Echocardiographic parameters of the study group

	Skin AF ≤2.2	Skin AF >2.2	P value
LVEF (%)	68.80 ± 7.20	65.82 ± 8.11	0.02
LA diameter (mm)	35.34 ± 4.01	38.91 ± 4.72	0.01
E (cm/s)	74.88 ± 15.62	74.37 ± 19.16	0.86
A (cm/s)	87.94 ± 22.28	93.80 ± 20.60	0.11
E/A ratio	0.89 ± 0.26	0.82 ± 0.24	0.11
E’(SW) (cm/s)	7.54 ± 2.13	6.17 ± 1.84	0.01
A’(SW) (cm/s)	10.11 ± 2.08	9.44 ± 2.10	0.06
E/E’ ratio	10.73 ± 3.81	12.90 ± 4.50	0.01
DT (ms)	212.44 ± 42.20	227.85 ± 51.90	0.06
LVMI (g/m^2.7^)	47.72 ± 11.62	61.22 ± 17.76	0.01
oLVM/pLVM	1.21 ± 0.21	1.62 ± 0.38	0.01

*Skin AF* skin autofluorescence, *LVEF* left ventricular ejection fraction, *LA* left atrium, *E* early diastolic peak velocity, *A* late diastolic peak velocity, *E′* early diastolic peak velocity at septal portion of mitral annulus, *A′* late diastolic peak velocity at septal portion of mitral annulus, *SW* septal wall, *DT* deceleration time, *LVMI* left ventricular mass index, *oLVM* observed left ventricular mass, *pLVM* predicted left ventricular mass

### Skin autofluorescence as an independent factor for appropriateness of left ventricular mass

Results from the univariate linear regression analysis investigating factors associated with LVMI are displayed in Additional file 1. Age, BMI, skin AF, SBP, PP, smoking, stroke, IHD, PAOD, eGFR, LA diameter, A, E/A ratio, E′, A′, E/E′ were associated with LVMI. Stepwise multivariate linear regression analysis revealed that skin AF was significantly associated with LVMI, independent from other covariates including PAOD, BMI, PP, A′, and eGFR. An increase of 1 arbitrary unit of skin AF explained an increase of 10.67 g/m^2.7^ of LVMI (Table 3).

**Table 3:** Stepwise multivariate linear regression analysis to investigate independent factors associated with (a) increased left ventricular mass and (b) observed left ventricular mass/predicted left ventricular mass

	Unstandardized coefficient B	SE	Standardized coefficient β	P value
(a)				
Skin AF (A.U.)	10.67	2.32	0.32	<0.01
PAOD	31.35	9.54	0.23	<0.01
BMI	1.16	0.32	0.25	<0.01
PP	0.23	0.09	0.19	<0.01
A′	−1.61	0.55	−0.21	<0.01
eGFR	−0.14	0.06	−0.17	0.02
(b)				
Skin AF (A.U.)	0.30	0.05	0.41	<0.01
A′	−0.04	0.01	−0.24	<0.01
LVEF	−0.01	0.003	−0.20	<0.01
PAOD	0.53	0.21	0.17	0.01
E/A	−0.23	0.10	−0.16	0.03

*SE*. standard error, *Skin AF* skin autofluorescence, *A.U*. arbitrary unit, *PAOD* peripheral arterial occlusive disease, *BMI* body mass index, *PP* pulse pressure, *A′* late diastolic peak velocity at septal portion of mitral annulus, *GFR* glomerular filtration rate, *LVEF* left ventricular ejection fraction, *E* early diastolic peak velocity, *A* late diastolic peak velocity

A further investigation to determine factors associated with the ratio of oLVM indexed to the pLVM is summarized in Additional file 2. Age, skin AF, DBP, smoking, IHD, PAOD, eGFR, LVEF, LA diameter, A, E/A, E′, A′, and E/E′ were associated with the ratio of oLVM indexed to pLVM. Stepwise multivariate linear regression analysis revealed that skin AF was significantly associated with the ratio of oLVM indexed to pLVM, independent from A′, LVEF, PAOD and E/A. An increase of 1 arbitrary unit of skin AF explained an increase of 0.3 of the ratio of oLVM indexed to pLVM.

### Skin AF as a useful tool for predicting LVH and inappropriate LVM

**Fig. 2:**
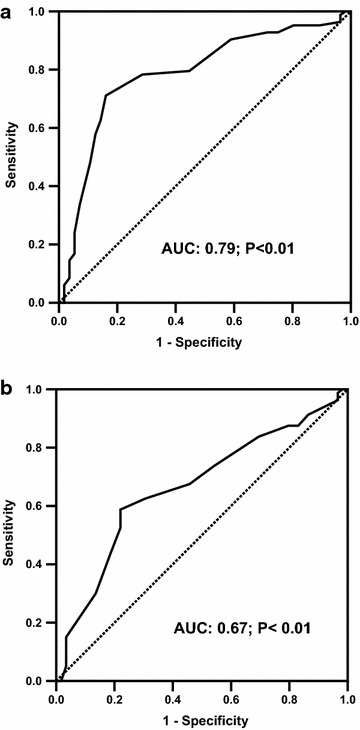
Receiver operating curves (ROC) of skin autofluorescence (AF) for predicting **a** inappropriate left ventricular mass and **b** left ventricular hypertrophy are displayed

The receiver operating characteristic (ROC) curve was used to determine the optimal cut-off value for predicting inappropriate LVM (Fig. 2a) and LVH (Fig. 2b). Skin AF ≥2.25 AU predicted inappropriate LVM at a sensitivity of 71.1%, and a specificity of 83.9% with an AUC of 0.79 (95% CI 0.71–0.87, P < 0.01). Skin AF ≥2.35 AU predicted LVH at a sensitivity of 58.8%, and a specificity of 73.0% with an area under the curve (AUC) of 0.67 (95% CI 0.57–0.76, P < 0.01).

### Association between skin AF and myocardial performance

**Fig. 3:**
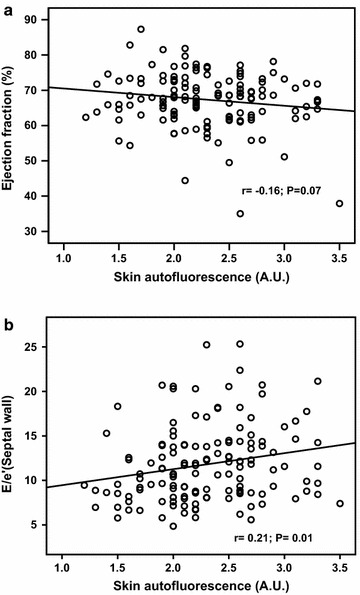
Correlation between skin autofluorescence and echocardiographic functional parameters are analyzed and displayed. **a** Skin autofluorescence is not significantly correlated with left ventricular systolic function. **b** Skin autofluorescence is negatively correlated with E/e′ ratio, a marker for diastolic function

We further tested whether measurements of skin AGEs accumulation could be associated with myocardial performance. In Fig. 3a and b, skin AF is associated with E/E′ (r = 0.21; P = 0.01), but not with LVEF (r = −0.16; P = 0.07).

### Relation between LVM and myocardial performance

In Table 4, both increased LVM and ratio of oLVM indexed to pLVM are significantly correlated with reduced E/A ratio, E′, A′ and increased LA diameter, E/E′, indicative of diastolic dysfunction. Only increased ratio of oLVM indexed to pLVM is correlated with reduced LVEF, indicative of systolic dysfunction.

**Table 4:** Correlation analysis between oLVM/pLVM ratio, LVMI, and echocardiographic parameters

	oLVM/pLVM		LVMI	
	r	P value	r	P value
LVEF	−0.28	<0.01	−0.13	0.12
LA diameter	0.31	<0.01	0.33	<0.01
E	−0.03	0.72	−0.03	0.74
A	0.21	0.01	0.18	0.03
E/A	−0.20	0.02	−0.20	0.02
E′	−0.32	<0.01	−0.33	<0.01
A′	−0.31	<0.01	−0.24	<0.01
E/E′	0.29	<0.01	0.29	<0.01
DT	0.16	0.06	0.12	0.17

*oLVM/pLVM* observed left ventricular mass/predicted left ventricular mass, *LVMI* left ventricular mass index, *LVEF* left ventricular ejection fraction, *LA* left atrium, *E* early diastolic peak velocity, *A* late diastolic peak velocity, *E′* early diastolic peak velocity at septal portion of mitral annulus, *A*′ late diastolic peak velocity at septal portion of mitral annulus, *DT* deceleration time

### Comparisons between appropriate LVM group and inappropriate LVM group

**Fig. 4:**
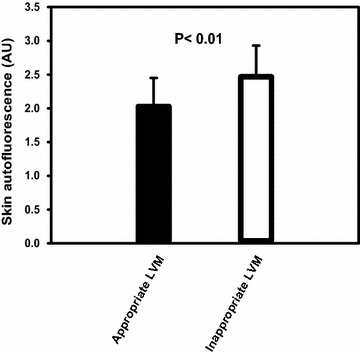
Comparisons of skin autofluorescence values between the appropriate left ventricular mass and inappropriate left ventricular mass are analyzed and depicted

Comparisons of baseline demographic and echocardiographic parameters between appropriate LVM and inappropriate LVM groups were analyzed, and the result is displayed in Table 5. Compared to the appropriate LVM, the inappropriate LVM has significantly higher proportions of female sex, IHD, and increased skin AF, LVMI, LA diameter, E/E′. In addition, the inappropriate LVM has significantly lower DBP, LVEF, E′, and A′, than the appropriate LVM. Difference in measured skin AF values between the two groups was further depicted in Fig. 4.

**Table 5:** Comparisons of baseline demographics and echocardiographic parameters between appropriate and inappropriate left ventricular mass

	Appropriate LVM (n = 56)	Inappropriate LVM (n = 83)	P value
Age (years)	61.73 ± 11.55	65.36 ± 9.16	0.052
Gender (M/F)	40/16	44/39	0.03
Skin AF (A.U.)	2.03 ± 0.42	2.47 ± 0.46	<0.01
BMI (kg/m^2^)	26.57 ± 3.61	26.09 ± 3.55	0.44
SBP (mmHg)	143.59 ± 13.66	138.82 ± 17.00	0.08
DBP (mmHg)	81.60 ± 12.72	75.84 ± 10.98	<0.01
PP (mmHg)	62.62 ± 14.42	63.10 ± 13.22	0.84
Hypertension n (%)	49 (87.50)	67 (80.72)	0.29
DM, n (%)			0.20
None, n (%)	36 (64.29)	54 (65.06)	
OHA, n (%)	19 (33.93)	22 (26.51)	
RI, n (%)	1 (1.78)	7 (8.43)	
Hyperlipidemia, n (%)	41 (73.21)	68 (81.92)	0.22
Stroke, n (%)	2 (3.57)	8 (9.64)	0.18
IHD, n (%)	7 (12.50)	23 (27.71)	0.03
PAOD, n (%)	0 (0)	2 (2.41)	0.24
LVMI (g/m^2.7^)	44.56 ± 9.28	60.91 ± 16.83	<0.01
eGFR (mL/min/1.73 m^2^)	67.17 ± 15.76	62.53 ± 22.92	0.16
Antiplatelets, n (%)	35 (62.50)	56 (67.47)	0.55
β-Blockers, n (%)	21 (37.50)	38 (45.78)	0.33
CCBs, n (%)	28 (50.00)	34 (40.96)	0.29
ACEIs/ARBs, n (%)	42 (75.00)	50 (60.24)	0.07
Statins, n (%)	30 (53.57)	51 (61.45)	0.36
Smoking			0.21
Never, n (%)	43 (76.79)	52 (62.65)	
Ever, n (%)	9 (16.07)	20 (24.10)	
Active, n (%)	4 (7.14)	11 (13.25)	
LVEF (%)	69.27 ± 8.16	66.04 ± 7.26	0.02
LA diameter (mm)	35.78 ± 4.20	37.98 ± 4.85	<0.01
E (cm/s)	73.31 ± 14.73	75.52 ± 18.99	0.47
A (cm/s)	88.13 ± 17.26	92.61 ± 24.02	0.23
E/A ratio	0.86 ± 0.26	0.85 ± 0.25	0.80
E′ (SW) (cm/s)	7.44 ± 2.11	6.49 ± 2.02	<0.01
A′ (SW) (cm/s)	10.35 ± 1.93	9.40 ± 2.15	<0.01
E/E′ ratio	10.65 ± 3.76	12.56 ± 4.47	<0.01
DT (ms)	220.09 ± 38.58	219.91 ± 53.14	0.98

*LVM* left ventricular mass, *M* male, *F* female, *Skin AF* skin autofluorescence, *A.U*. arbitrary unit, *BMI* body mass index, *SBP* systolic blood pressure, *DBP* diastolic blood pressure, *PP* pulse pressure, *DM* diabetes mellitus, *OHA* oral hypoglycemic agent, *RI* regular insulin, *IHD* Ischemic heart disease, *PAOD* peripheral arterial occlusive disease, *LVMI* left ventricular mass index, *GFR* glomerular filtration rate, *CCBs* calcium channel blockers, *ACEIs* angiotensinogen converting enzyme inhbitors, *ARBs* angiotensin II type I receptor blockers, *LVEF* left ventricular ejection fraction, *LA* left atrium, *E* early diastolic peak velocity, *A* late diastolic peak velocity, *E′* early diastolic peak velocity at septal portion of mitral annulus, *A′* late diastolic peak velocity at septal portion of mitral annulus, *SW* septal wall, *DT* deceleration time

Considering the potential confounding effect of LVH, we further divided the study subjects into two subgroups according to the presence of LVH. The result is displayed in Table 6. In subjects without LVH, an increase in the ratio of oLVM/pLVM shows a trend, though not statistically significant, toward a reduced LVEF.

**Table 6:** Correlation analysis between oLVM/pLVM ratio and echocardiographic parameters stratified according to the presence of LVH

	Non-LVH (n = 59)		LVH (n = 80)	
	r	P value	r	P value
LVEF	−0.24	0.06	−0.34	<0.01
LA diameter	0.11	0.39	0.24	0.03
E	−0.08	0.55	0.01	0.97
A	0.34	<0.01	0.02	0.85
E/A	−0.23	0.08	−0.01	0.90
E′	−0.18	0.17	−0.25	0.03
A′	−0.15	0.27	−0.32	<0.01
E/E′	0.10	0.46	0.27	0.02
DT	−0.03	0.81	0.13	0.26

*oLVM/pLVM* observed left ventricular mass/predicted left ventricular mass, *LVH* left ventricular hypertrophy, *LVEF* left ventricular ejection fraction, *LA* left atrium, *E* early diastolic peak velocity, *A* late diastolic peak velocity, *E′* early diastolic peak velocity at septal portion of mitral annulus, *A*′ late diastolic peak velocity at septal portion of mitral annulus, *DT* deceleration time

## Discussion

In this study, we proposed that skin AF could predict LVH and inappropriate LVM. In addition, increased skin AF value is associated with elevated mitral filling pressure, a marker for diastolic dysfunction.

### Skin AF as a marker for subclinical microvascular and macrovascular diseases

The application of skin AF as a marker for microvascular and macrovascular diseases in subjects with diabetes or CKD has been proposed. Cho et al. proposed significantly higher skin AF in young adolescents of type 1 diabetes with retinopathy than those without retinopathy [18]. Higher skin AF associated with endothelial dysfunction [19] and arterial pulse wave velocity [20] has been described in subjects with end-stage renal failure. Recently, Sell et al. proposed skin collagen fluorophore LW-1 to be a useful marker for the subclinical macrovascular disease in patients with type 1 diabetes. They suggested that LW-1 level is significantly correlated with skin intrinsic fluorescence (SIF) level, a surrogate marker for tissue AGEs accumulation, and higher skin LW-1 level is associated with increased intima-media thickness (IMT) and LVM. However, in their study, the SIF level is not significantly correlated with LVM, which is contradictory to our result [21]. The discrepancy is possibly explained by the different methods of measuring tissue AGEs accumulation, calculating LVM, and different study populations with respect to ethnicity, age-distribution, and baseline comorbidities.

### Rationale of tissue AGEs accumulation in relation to inappropriate LVM

Previous studies have suggested that chronic kidney disease is associated with LVH and inappropriate LVM [22–25]. However, the possible explanation for this association is not clear. AGEs are increased in subjects with chronic kidney disease due to increased oxidative stress, and decreased renal excretion [26]. A small scale clinical trial also observed that treatment with AGEs breaker may improve left ventricular diastolic function and LVM regression [27]. Therefore, we hypothesized that AGEs might be related to LVM growth. Makulska et al. [28] investigated the association between skin AF and cardiovascular risk and proposed that skin AF was significantly correlated with LVM in children with chronic kidney disease stage 2–5. However, whether the increased LVM is inappropriate is not clear. Our study suggest that the amount of skin AGEs deposition is not only associated with LVH, but also with the extent of LVM growth exceeding its physiological need.

### Factors determining left ventricular mass

Increased hemodynamic load, body size, and gender are key factors for LVM growth. The adaptive response is used to protect myocardium from excessive wall stress, providing sufficient nutrients to body tissue by maintaining or increasing cardiac output [29]. However, in some cases, the LVM may grow beyond the compensatory need and become inappropriate. This indicates that other factors may be involved in the abnormal LVM growth, such as genetic polymorphism [30], neurohormones [31], and cytokine [32]. Our study suggests that AGEs are related to inappropriate LVM. AGE-stimulated ECM gene expression and deposition might partially explain its association with abnormally increased LVM [9].

### Higher predictive value of skin AF in detecting inappropriate LVM than detecting LVH

Our study demonstrated that skin AF had higher sensitivity and specificity in detecting inappropriate LVM than in detecting LVH. In our study, over eighty percent of the study population have chronic hypertension. Therefore, some LVH may be an appropriate response to the increased hemodynamic load.

### Tissue AGEs accumulation in relation to impaired myocardial performance

In Table 2, increased skin AF value is associated with increased LA diameter, elevated E/E′, and reduced E′, indicating increased LA pressure and impaired relaxation. This suggests that skin AF could be a useful marker to detect diastolic dysfunction. At least 3 possible explanations to support this idea: first, AGEs may cross-link with type IV collagen, elastin to increase ventricular stiffness; second, AGEs may impair myocardial calcium handling and lead to diastolic dysfunction [33]; third, AGEs may stimulate the excretion of TGF-β, thus increasing the ECM [9], and myocardial fibrosis. However, our data suggests that skin AF value is not associated with LVEF. This required further evaluation. A previous study has proposed that treatment with AGEs breaker for 36 weeks can neither improve clinical symptoms, nor myocardial function in the setting of systolic HF [34]. Our findings are in line with previous observations that tissue AGEs deposition may influence myocardial diastolic performance, rather than systolic dysfunction.

### Association between inappropriate LVM and myocardial dysfunction

In Table 4, an increased ratio of oLVM/pLVM is associated with decreased LVEF, E/A ratio, E′, A′, and increased, E/E′. This implies that there is a relationship between inappropriate LVM and cardiac functional abnormalities, a finding accords with previous findings [35–37].

In Table 6, the ratio of oLVM/pLVM shows a trend toward an inverse association with LVEF in the setting of non-LVH subgroup. Mureddu et al. proposed that in the setting of chronic hypertension, subjects without LVH, but with inappropriate LVM, have a significantly lower cardiac index, and reduced left ventricular mid-wall shortening compared to subjects with appropriate LVM [38]. Our data correspond to their findings. The MAVI study indicated that in the non-LVH subgroup analysis, subjects with inappropriate LVM still had a worse prognosis compared with subjects with appropriate LVM [1]. The inappropriate LVM-related cardiac functional abnormalities may provide some explanations.

### Study limitations

First, because this is an observational case–control study, we could only establish an association between tissue AGEs accumulation and inappropriate LVM. We could not provide any evidence of causal effect. Second, because we only enrolled subjects with a moderate to high risk for cardiovascular diseases, our study results cannot be generalized to other populations. Third, although we measured the subjects’ blood pressure before the echocardiography study in order to calculate the predicted LVM, this single “office” blood pressure value may not represent the chronic wall stress imposed on the left ventricle. Whether ambulatory blood pressure monitoring could be more representative requires further validation. Fourth, skin AF could only estimate the amount of tissue AGEs with fluorescent properties. Those without fluorescent properties could not be measured by skin AF. However, a previous study has established an association between skin AF and the amounts of skin deposition of both fluorescent and non-fluorescent AGEs [11]. Fifth, low vitamin D status has been linked to left ventricular hypertrophy [39], and elevated skin AF is associated with low serum vitamin D level in subjects with type 2 DM [40]. In our study, we cannot provide the subjects’ serum vitamin D level, which could be a potential confounding factor. Sixth, we used skin AF value as a surrogate marker for skin AGEs accumulation. We did not provide an evidence that skin AF value could reflect the amount of myocardial AGEs deposit. However, previous study has reported a close association between skin AF values and tissue AGEs levels of atrial appendage [41].

## Conclusions

We proposed that skin AF value is a useful marker for predicting LVH, and inappropriate LVM and diastolic dysfunction in subjects at risk for cardiovascular diseases. Tissue AGEs accumulation might play some role in LVM growth exceeding its physiological need.

## Additional files

**Additional file 1.** Univariate linear regression analysis for the association between left ventricular mass index and other factors.

**Additional file 2.** Univariate linear regression analysis for the association between appropriateness of left ventricular mass and other factors.

## Abbreviations

LVM: left ventricular mass
CV: cardiovascular
AGEs: advanced glycation end products
ECM: extracellular matrix
AF: autofluorescence
LVH: left ventricular hypertrophy
DM: diabetes mellitus
IHD: ischemic heart disease
PAOD: peripheral arterial occlusive disease
BMI: body mass index
eGFR: estimated glomerular filtration rate
MDRD: modification of diet in renal disease
ABI: ankle-brachial index
LVID: left ventricular internal diameter
SWT: septal wall thickness
PWT: posterior wall thickness
BSA: body surface area
EDV: left ventricular volume at end diastolic phase
ESV: left ventricular volume at end-systolic phase
LVEF: left ventricular ejection fraction
SW: stroke work
DT: deceleration time
AU: arbitrary units
ROC: receiver operating characteristic curve
LA: left atrium
AUC: an area under the curve
