# Supplementary material for: Skin autofluorescence is associated with inappropriate left ventricular mass and diastolic dysfunction in subjects at risk for cardiovascular disease
Source: Cardiovasc Diabetol. 2017 Jan 25;16:15. doi: 10.1186/s12933-017-0495-9 (PMC5267439; doi:10.1186/s12933-017-0495-9)
Supplement: Supplementary file 1 — Additional file 1. Univariate linear regression analysis for the association between left ventricular mass index and other factors. [file 12933_2017_495_MOESM1_ESM.docx]

|  | Unstandardized  coefficient B | SE | Standardized coefficienct β | P value |
| --- | --- | --- | --- | --- |
| Age | 0.29 | 0.13 | 0.19 | 0.03 |
| Gender (F vs M) | 0.79 | 2.85 | 0.02 | 0.78 |
| BMI | 0.83 | 0.39 | 0.18 | 0.03 |
| Skin AF | 11.92 | 2.64 | 0.36 | <0.01 |
| SBP | 0.21 | 0.09 | 0.21 | 0.02 |
| DBP | -0.11 | 0.12 | -0.08 | 0.36 |
| PP | 0.36 | 0.10 | 0.30 | <0.01 |
| Smoking | 3.46 | 2.03 | 0.14 | 0.09 |
| DM | 1.89 | 2.32 | 0.07 | 0.42 |
| HTN | 6.82 | 3.70 | 0.16 | 0.07 |
| Hyperlipidemia | 1.84 | 3.38 | 0.05 | 0.59 |
| Stroke | 11.67 | 5.30 | 0.19 | 0.03 |
| IHD | 9.18 | 3.29 | 0.23 | <0.01 |
| PAOD | 37.57 | 11.24 | 0.28 | <0.01 |
| eGFR | -0.24 | 0.07 | -0.29 | <0.01 |
| Antiplatelets | -3.80 | 2.91 | -0.11 | 0.19 |
| β-blockers | 2.66 | 2.81 | 0.08 | 0.35 |
| CCBs | 2.70 | 2.79 | 0.08 | 0.34 |
| ACEIs/ARBs | 4.75 | 2.92 | 0.14 | 0.11 |
| Statins | -0.66 | 2.82 | -0.02 | 0.82 |
| LVEF | -0.28 | 0.18 | -0.13 | 0.12 |
| LA diameter | 1.13 | 0.28 | 0.33 | <0.01 |
| E | -0.03 | 0.08 | -0.03 | 0.74 |
| A | 0.14 | 0.06 | 0.18 | 0.03 |
| E/A ratio | -13.26 | 5.46 | -0.20 | 0.02 |
| E’ | -2.57 | 0.63 | -0.33 | <0.01 |
| A’ | -1.84 | 0.64 | -0.24 | <0.01 |
| E/E’ | 1.10 | 0.31 | 0.29 | <0.01 |
| DT | 0.04 | 0.03 | 0.12 | 0.17 |
|  |  |  |  |  |

Table S1 Univariate linear regression analysis for the association

between left ventricular mass index and other factors

SE: Standard error; F: female; M: male; BMI: Body mass index; Skin AF: Skin autofluorescence; SBP: Systolic blood pressure; DBP: Diastolic blood pressure; PP: Pulse pressure; DM: Diabetes mellitus; HTN: Hypertension; IHD: Ischemic heart disease; PAOD: Peripheral arterial occlusive disease, eGFR: estimated glomerular filtration rate; CCBs: Calcium channel blockers; ACEIs: Angiotensin-converting enzyme inhibitors; ARBs: Angiotensin II receptor blockers; LVEF: Left ventricular ejection fraction; LA: Left atrium; DT: Deceleration time
