# Supplementary material for: Skin autofluorescence is associated with inappropriate left ventricular mass and diastolic dysfunction in subjects at risk for cardiovascular disease
Source: Cardiovasc Diabetol. 2017 Jan 25;16:15. doi: 10.1186/s12933-017-0495-9 (PMC5267439; doi:10.1186/s12933-017-0495-9)
Supplement: Supplementary file 2 — Additional file 2. Univariate linear regression analysis for the association between appropriateness of left ventricular mass and other factors. [file 12933_2017_495_MOESM2_ESM.docx]

|  | Unstandardized  coefficient B | SE | Standardized coefficient β | P value |
| --- | --- | --- | --- | --- |
| Age | 0.006 | 0.003 | 0.18 | 0.04 |
| Gender (F vs M) | 0.02 | 0.06 | 0.03 | 0.73 |
| BMI | -0.001 | 0.009 | -0.008 | 0.92 |
| Skin AF | 0.38 | 0.05 | 0.51 | <0.01 |
| SBP | -0.003 | 0.002 | -0.12 | 0.17 |
| DBP | -0.006 | 0.003 | -0.18 | 0.03 |
| PP | 0.00 | 0.002 | 0.01 | 0.88 |
| Smoking | 0.11 | 0.05 | 0.21 | 0.01 |
| DM | 0.007 | 0.05 | 0.01 | 0.89 |
| HTN | -0.02 | 0.08 | -0.02 | 0.80 |
| Hyperlipidemia | -0.04 | 0.08 | -0.04 | 0.65 |
| Stroke | 0.16 | 0.12 | 0.11 | 0.19 |
| IHD | 0.23 | 0.07 | 0.26 | <0.01 |
| PAOD | 0.66 | 0.26 | 0.22 | 0.01 |
| eGFR | -0.004 | 0.002 | -0.21 | 0.01 |
| Antiplatelets | 0.06 | 0.07 | 0.07 | 0.39 |
| β-blockers | 0.09 | 0.06 | 0.13 | 0.14 |
| CCBs | 0.002 | 0.06 | 0.003 | 0.97 |
| ACEIs/ARBs | -0.12 | 0.07 | -0.16 | 0.07 |
| Statins | 0.00 | 0.06 | 0.00 | 1.00 |
| LVEF | -0.01 | 0.004 | -0.28 | <0.01 |
| LA diameter | 0.02 | 0.01 | 0.31 | <0.01 |
| E | -0.001 | 0.002 | -0.03 | 0.72 |
| A | 0.004 | 0.001 | 0.21 | 0.01 |
| E/A | -0.29 | 0.12 | -0.20 | 0.02 |
| E’ | -0.06 | 0.01 | -0.32 | <0.01 |
| A’ | -0.05 | 0.01 | -0.31 | <0.01 |
| E/E’ | 0.03 | 0.01 | 0.29 | <0.01 |
| DT | 0.001 | 0.001 | 0.16 | 0.06 |

Table S2 Univariate linear regression analysis for the association between appropriateness of left ventricular mass and other factors

SE: Standard error; F: female; M: male; BMI: Body mass index; Skin AF: Skin autofluorescence; SBP: Systolic blood pressure; DBP: Diastolic blood pressure; PP: Pulse pressure; DM: Diabetes mellitus; HTN: Hypertension; IHD: Ischemic heart disease; PAOD: Peripheral arterial occlusive disease, eGFR: estimated glomerular filtration rate; CCBs: Calcium channel blockers; ACEIs: Angiotensin-converting enzyme inhibitors; ARBs: Angiotensin II receptor blockers; LVEF: Left ventricular ejection fraction; LA: Left atrium; DT: Deceleration time
